# New Technique for Tibiotalar Arthrodesis Using a New Intramedullary Nail Device: A Cadaveric Study

**DOI:** 10.1155/2016/5247647

**Published:** 2016-10-13

**Authors:** Emmanuel D. Eisenstein, Mario Rodriguez, Amr A. Abdelgawad

**Affiliations:** ^1^Texas Tech University Health Sciences Center, El Paso, TX, USA; ^2^Department of Orthopaedic Surgery and Rehabilitation, William Beaumont Army Medical Center, El Paso, TX, USA; ^3^Merdo Innovation and Engineering, LLC, El Paso, TX, USA

## Abstract

*Introduction*. Ankle arthrodesis is performed in a variety of methods. We propose a new technique for tibiotalar arthrodesis using a newly designed intramedullary nail.* Methods*. We proposed development of an intramedullary device for ankle arthrodesis which spared the subtalar joint using a sinus tarsi approach. Standard saw bones models and computer assisted modeling and stress analysis were used to develop different nail design geometries and determine the feasibility of insertion. After the final design was constructed, the device was tested on three cadaveric specimens.* Results*. Four basic nail geometries were developed. The optimal design was composed of two relatively straight segments, each with a different radius of curvature for their respective tibial and talar component. We successfully implemented this design into three cadaveric specimens.* Conclusion*. Our newly designed tibiotalar nail provides a new technique for isolated tibiotalar fusion. It utilizes the advantages of a tibiotalar calcaneal nail and spares the subtalar joint. This design serves as the foundation for future research to include compression options across the tibiotalar joint and eventual transition to clinical practice.

## 1. Introduction

Ankle arthrodesis has been reliably used and improved since 1951 when Charnley reported his method of compression arthrodesis with the use of external fixation [1]. Damage to the ankle can be due to a variety of reasons such as trauma or pathology which causes progressive loss and damage to the articular surface. Arthrodesis is used for symptomatic, persistent, disabling ankle pain refractory to nonoperative management to include NSAIDs, shoe modifications, or bracing [1]. Despite improvement in approaches and methods of fixation, limitations still exist for the current treatment strategies available. Open techniques with the use of plates and screws can be associated with soft tissue stripping which is not biologically sound for bony healing, while less invasive techniques such as the concomitant use of arthroscopy or tibiotalarcalcaneal (TTC) intramedullary nailing do not allow for correction of severe deformity and in the case of TTC nailing involve the subtalar joint which may not be diseased [2, 3]. We propose a new technique using a newly developed tibiotalar intramedullary device via an extended sinus tarsi approach which combines the advantages of current techniques and decreases their limitations.

*Current Techniques*. Current methods for ankle arthrodesis include open, mini open, and combined methods including the use of arthroscopy for joint preparation or external fixation for fusion. External fixation combines an open surgical preparation of the joint with external fixation. This method is usually recommended for osteopenic patients with suboptimal bone quality or those with preexisting septic arthritis which preclude the use of internal fixation [1, 4]. This is a viable option compared to amputation in patients with severe infection as a salvage procedure [2, 4]. This method has been associated with increased stiffness in bending and torsion with comparable compressive strength when compared to crossed screws [5]. Circular external fixation also adds the benefit of earlier weight-bearing [4].

Arthroscopic and mini open ankle arthrodesis involves joint preparation through arthroscopic means, or, in the case of mini open techniques, the joint preparation is done with the use of enlarged portals. The fusion procedure is then carried out with the use of screws or screws and plates, with the use of screws along with plates offering increased stability compared to screws alone [6, 7]. The decreased soft tissue stripping compared to open techniques has been reported to be associated with quicker fusion rates [1–3].

TTC nails offer the advantage of increased primary stiffness when compared to external fixation or the use of screws [8]. This approach is also associated with minimal periosteal and soft tissue stripping along with a minimally invasive approach.

*Current Techniques' Complications*. External fixation is associated with pin tract infections, the need for a secondary procedure for fixator removal, and residual angular deformities [2]. Arthroscopic and mini open procedures are not recommended for patients with ankle deformity and malalignment [1]. The use of screws alone has been shown to have decreased biomechanical strength when compared to plates or external fixation [5, 6]. TTC nails are contraindicated in patients with an intact subtalar joint, significant tibial deformity, or loss of calcaneal body height [2]. Malalignment is also common and is due to the inclusion of the subtalar joint as part of the construct [2]. Although TTC nails represent a minimally invasive technique, requiring a small plantar incision, the lateral plantar nerve and artery are at risk for injury at the insertion site [1].

## 2. Methods

### 2.1. New Device for Isolated Tibiotalar Arthrodesis

#### 2.1.1. Conceptualization of Approach/Insertion and Hardware

The intention of our design was to create an intramedullary device which spares the subtalar joint in contrast to the current implants available. As part of the first stage of implant design, we investigated previous intellectual work in the field by looking through the US patent list to determine whether any similar projects were being developed. We did not find any related patents to our concept of ankle arthrodesis using an intramedullary nail traversing only the talus and tibia secured by screw fixation. This device was developed to be inserted through a minimally invasive sinus tarsi approach with extension into an approach to the distal lateral fibula. Extension into the lateral distal fibula is required for distal fibula osteotomy and preparation of the tibiotalar joint and to provide local bone graft for fusion.

#### 2.1.2. Insertion/Approach Development

**Figure 1:**
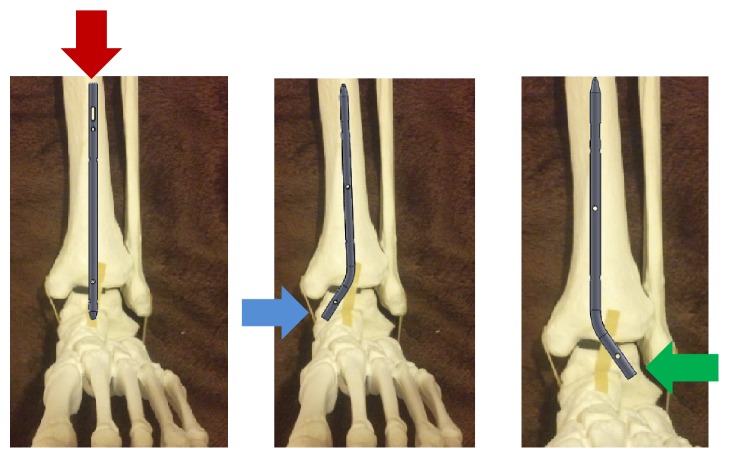
Insertion points.

**Figure 2:**
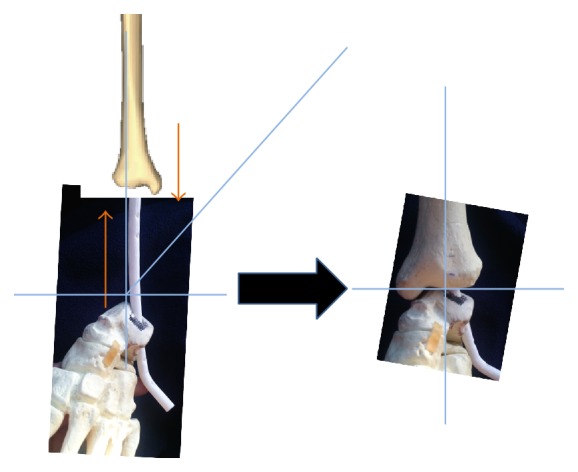
Varus angulation of 45 degrees required for nail insertion.

We postulated three different possible insertion points which would avoid the subtalar joint: a proximal to distal insertion from tibia to talus, a distal medial talus to tibia insertion, and a distal lateral talus to tibia insertion (Figure 1). We originally planned to use a minimally invasive sinus tarsi approach and thus proceeded with development of the nail with the goal of insertion to be from the lateral talus into the tibia without testing the other insertion points. This, however, created a concern for the talus to have sufficient lateral bone surface area to support the mechanical stress of weight-bearing. Another concern with this approach is the need to angulate the talus into varus, so the hole created in the talus lines up with the intramedullary canal of the tibia (Figure 2). This concern was mitigated by the fact that in the typical arthrodesis procedure a distal fibula osteotomy is created to allow preparation of the joint and to provide local bone graft.

**Figure 3:**
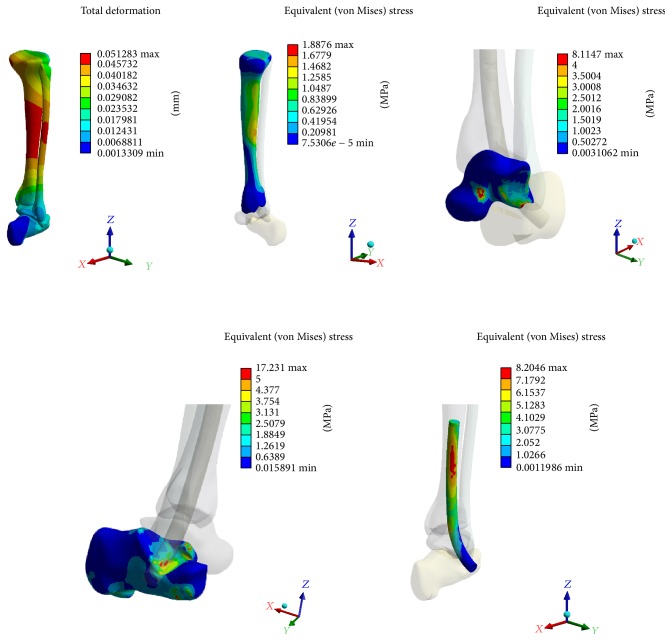
Computational stress analysis with high stress areas at the mid tiba and fibula, at the insertion point of the talus, and in the calcaneus at the area closest to the nail curvature. The nail itself sees the most stress in the proximal aspect.

Computational stress analysis was performed by applying an axial load of 350 N to the proximal tibia simulating weight-bearing of an average person. This analysis was performed to determine the areas of bone and implant susceptible to stress and narrow the choices as to which geometries and materials would best serve our requirements and confirm the ability of a lateral talus insertion point (Figure 3).

Our stress analysis revealed that the tibia and fibula see the most amount of stress around the midshaft; similarly, the nail itself is subjected to high stress at its proximal aspect as this area carries the axial load at first instance. The talus itself is under highest stress around the nail insertion site, while the calcaneus is also subject to stress around the nail curvature due to transfer of the talus insertion site to nearby areas. This analysis also suggests that the lateral talus wall would offer enough support for weight-bearing but actual load mechanical testing is necessary to confirm it. The titanium alloy Ti6aL4V ELI was chosen as a reasonable option for nail composition due to lower mechanical failure rates and improved biocompatibility compared to stainless steel.

#### 2.1.3. Nail Development

We started by creating a list of design specifications that the nail must achieve in order to be successfully implemented. It needed to be an intramedullary device of at least 8 mm to 10 mm in diameter (similar to currently available devices) of varying lengths of 20–35 cm, with proximal and distal locking screw options to prevent any movement, and must resist high axial loading forces as well as bending moments and torsion. The material itself should be suitable for human application (titanium or stainless steel) with easy surgical application and removal.

**Figure 4:**
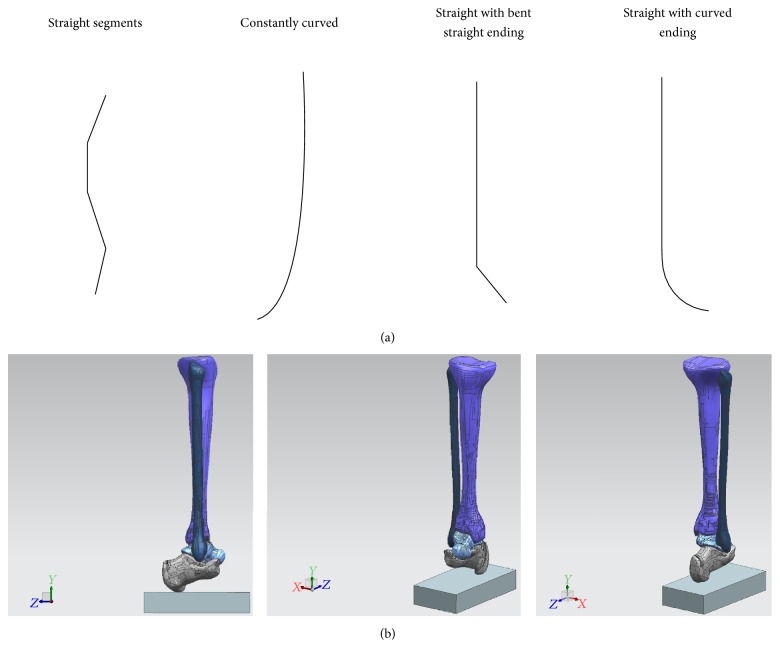
(a) Basic structure geometries. (b) CAD model.

With these ideas in mind, we then created a table in which we looked at all the TTC nails currently available and organized them by company, nail geometry, nail diameter, nail lengths, and nail material and created a second table looking at similar information for the locking screws. These would serve as a starting point for hardware development. We began by looking at 4 basic structure geometries: straight, constantly curved, straight with bent straight ending, and straight with curved ending (Figure 4(a)). In order to visualize and study the geometry of the nail, the different angle of curvature, and nail arrangements to meet our requirements of avoiding the subtalar joint while providing appropriate alignment for tibiotalar arthrodesis, we used computer-aided design (CAD) tools and a standard saw bones model of a tibia, fibula, talus, and calcaneus. For simulation purposes, a nail model of 10 mm in diameter was used (Figure 4(b)).

The straight segment configuration is composed of united multiple straight segments which have very high stress concentration at the interface of the multiple united segments. The constantly curved model distributes stresses more uniformly than the straight segment model but insertion may be difficult due to the curvature, while the straight with bent straight end segment allows easier insertion achieved with talus angulation and has a well carrying load design as there is only one union for the two segments. The fourth option, straight with curved ending, combines the advantages of the 2nd and 3rd models and reduces the stress concentration at the union point of the segments but makes insertion more difficult as it would require a higher diameter entry hole. Thus, we concentrated on developing the nail with options 2 and 3 as our basic geometry.

#### 2.1.4. Final Nail Design

**Figure 5:**
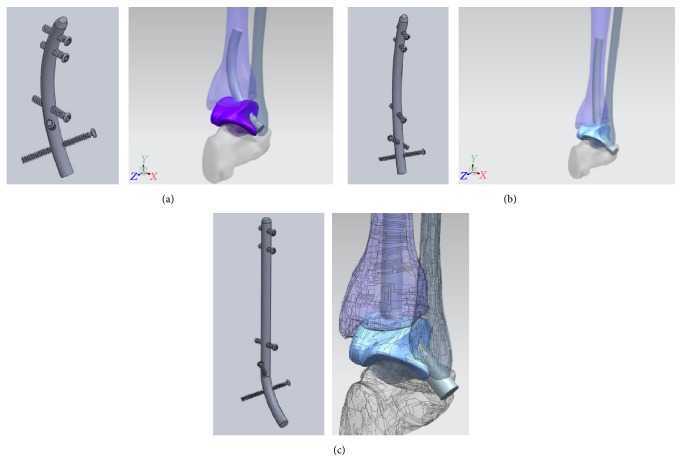
Constant curve nail with 1 curve (CC1), constant curve nail with 2 independent curves (CC2), and straight with bent straight end nail (SBSE).

We proposed 3 different models using the basic geometric constructs discussed. The first design is based on the constant curve nail geometry with one curvature (CC1) to pass through the talus and tibia (Figure 5(a)). The second design is also based on the constant curve geometry but with two different curves (CC2), one in the tibia segment and one in the talus segment (Figure 5(b)). The third design is based on the straight with bent straight ending (SBSE) segment (Figure 5(c)).

**Figure 6:**
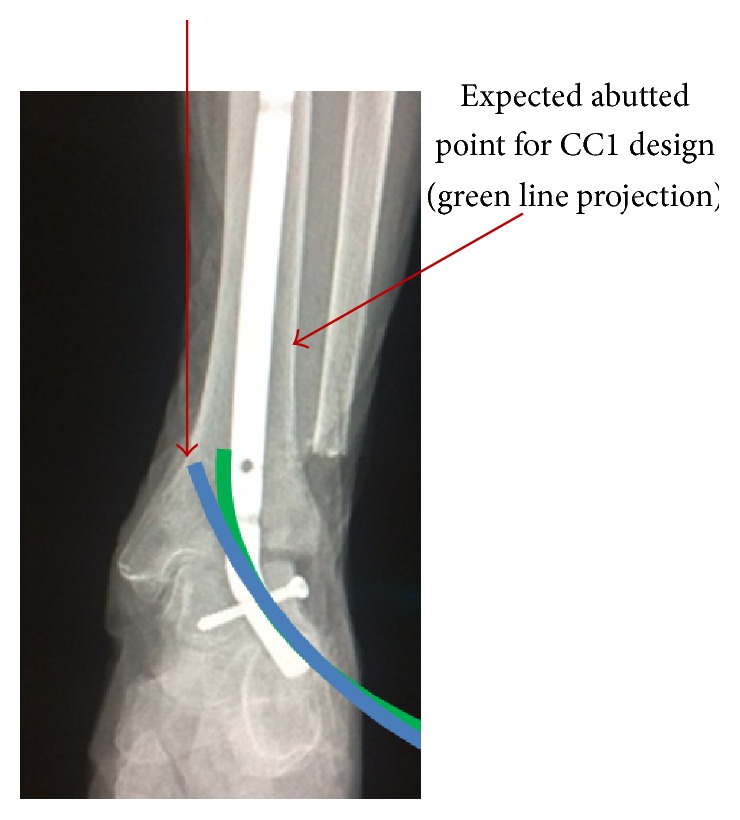
Green line represents current radius of curvature proposed with approximate abutment in lateral tibia; blue line represents larger radius of curvature theoretically allowing a longer implant in the tibial segment; however, it abuts the medial tibia instead of progressing proximally.

The CC1 design provides the advantage of not needing to angulate the talus during insertion; however, a curved hole is required for insertion, and thus a curved reamer would be necessary which obviates its main advantage. It is also limited in the inability to increase the length of the nail for the purposes of increased stability as the curvature of the nail abuts the tibia at a relatively short distance of 13 cm with a 12.79 cm radius of curvature at an angle of 16.58 degrees. These measurements were done based on the standard sawbones model of the lower extremity and allow for the longest tibial segment possible. While increasing the radius of curvature would theoretically allow placement of a longer tibial segment, the nail would abut the medial wall of the tibia instead of progressing proximally. The short distance into the tibial intramedullary canal limiting the stability of the construct along with the need for a curved reamer which requires a longer tunnel and increased bone loss compared to a straight tunnel for insertion limits this nail design (Figure 6).

**Figure 7:**
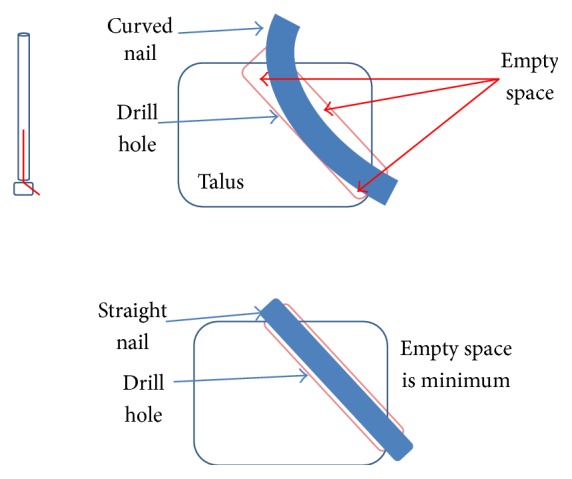
The more pronounced curvature of the talus segment due to the smaller radius of curvature requires a larger diameter drill hole for insertion, thus removing more bone compared to a straight design.

The CC2 design has two separate curvatures, each designed for the respective tibial and talar segment. Having two segments with different curvatures allows for an increased insertion length of the tibial segment. This design was thought to increase the insertion distance of the nail into the tibia with a greater radius of curvature in the tibial segment to gain distance into the intramedullary canal combined with a smaller radius of curvature in the talar segment. This would allow insertion without the need for angulation, simultaneously avoiding the subtalar joint while increasing the tibial segment length. However, the smaller radius of curvature in the nail for the talus segment requires a larger diameter drill hole in the bone to support curved nail insertion. By increasing the drill hole diameter, the amount of bone removed from talus also increases and affects the mechanical stability in the talus, as there is less bone mass to support stresses (Figure 7). Due to the increased talar bone loss required for insertion and the associated decreased mechanical stability this causes, this design was deemed inadequate.

**Figure 8:**
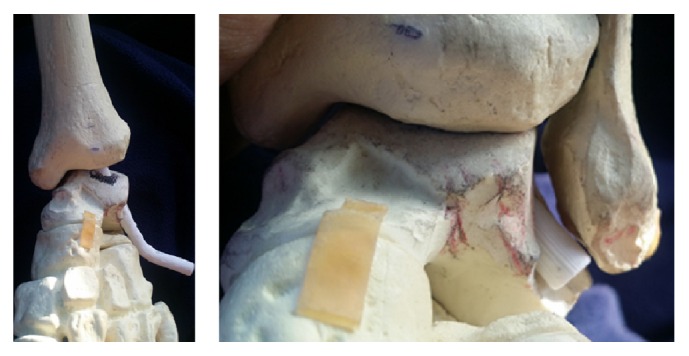
Varus angulation for insertion and normal tibiotalar relationship after full seating.

The SBSE design requires an estimated varus angulation of 37.24 degrees, based on the standard sawbones lower extremity model, during insertion and allows a normal tibiotalar relationship after fully seating the nail (Figure 8).

The straight segments of the nail help with screw insertion and increase stability by limiting the movement of the nail in the two bones as can be seen in Figure 7. The lack of empty space allows for a greater contact surface area thus the nail position is more stable, maintaining the appropriate plantar/dorsiflexion as well as varus/valgus angulation.

**Figure 9:**
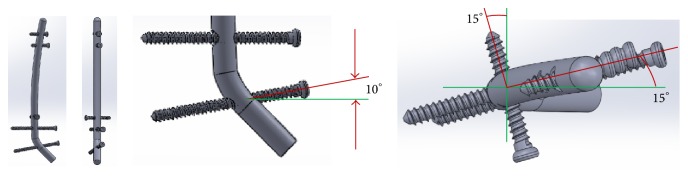
Final nail design.

The ability to angulate the talus into varus is facilitated by the standard fibular osteotomy and the design was modified slightly by adding a curvature to the talus segment to facilitate a deeper, more medial entry into the talus to allow for sufficient residual lateral wall to withstand the physiologic stresses of weight-bearing. The SBSE design is also able to increase its length due to the straight tibial segment. The SBSE design was then carried over as the basis of the final design with an added curve to the initially straight tibial segment (radius 524.15 mm), a talus segment curvature of 26.55 degrees, an 8–10 mm diameter, and five 4.0 mm locking screw options in multiple planes (Figure 9). These final curvatures allow for longer insertion into the tibia, minimize the amount of varus angulation required for insertion, and minimize the amount of talus bone loss during drilling, making use of the advantages and minimizing the shortcomings of all three designs.

## 3. Results

### 3.1. Cadaveric Application

**Figure 10:**
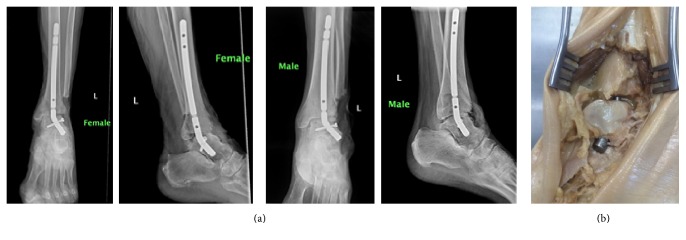
(a) AP and lateral radiographs with intramedullary device in place in appropriate coronal alignment. (b) Cadaveric specimen with intramedullary device flush in sinus tarsi.

After approval from the Willed Body Program at our university, we proceeded to test our device in three cadaveric specimens. We used two female and one male left lower extremities to assess the feasibility of insertion of the device as well as to test its ability to self-correct the tibiotalar joint into its normal tibiotalar relationship (the recommended five degrees of valgus as is the current practice when performing ankle fusion) [1]. We made a 10 cm incision encompassing the lateral approach to the distal fibula as well as the sinus tarsi. Our approach was much larger than required in order to fully visualize all structures during our initial trial insertion. After incising the skin and subcutaneous fat, we were able to visualize the fibula proximally and the fat pad within the sinus tarsi. We then excised the fat pad and detached the extensor digitorum brevis from the superolateral anterior process of the calcaneus in the standard fashion as part of the sinus tarsi approach. Proximally, at the fibula, we incised the periosteum directly on the anterior 1/3 of the fibula and created subperiosteal flaps making sure to stay anterior to the peroneal tendons and peroneal tendon sheath. Continuing with subperiosteal dissection distally and circumferentially around the fibula we then used an osteotome to excise the distal 4 cm of fibula. This allowed us to fully visualize the tibiotalar joint. We prepared the tibiotalar joint by denuding the cartilage surfaces using an osteotome. We then identified the lateral process of the talus as our landmark for entry and insertion of our newly developed tibiotalar intramedullary device. Using an 8 mm reamer, we proceeded to create a path for nail insertion through the inferior aspect of the lateral process into the talus and subsequently into the tibia. Initial varus deformity is required to be able to place the reamer into appropriate position just beneath the lateral process of the talus. In our first attempt, we failed to recognize the amount of medial direction that is required to ream into the body of the talus to allow for an appropriate amount of lateral wall for locking screw insertion and thus proceeded to breach the lateral wall of the talus. With the inferior aspect of the talus parallel to the floor as a reference, an angle of 40 degrees in the superomedial direction allows for appropriate insertion into the mid body of the talus. Our initial angle which failed was about 60 degrees from the horizontal plane, 20 degrees more lateral than necessary. This error confirmed the angulation of 37.24 degrees which was proposed during nail design. Once the reamer exited the proximal middle aspect of the talar body, we accentuated the varus deformity in order to aim the reamer towards the middle of the tibial intramedullary canal and reamed the distal 3 cm of tibia. We then proceeded to insert our intramedullary device with gentle blows from a hammer. During nail insertion, the varus deformity was self-corrected into the recommended 5 degrees of valgus for tibiotalar fusion (Figure 10(a)). The nail was then tapped until flush at the sinus tarsi, and a locking screw was inserted into the lateral wall of the talus (Figure 10(b)). After the initial breach of the lateral wall on female cadaver # 1, we had no difficulty replicating the required angulation and trajectory of the reamer on the 2nd female and the male cadaver.

## 4. Discussion

Current methods of tibiotalar arthrodesis are each associated with disadvantages. Our proposed technique incorporates the advantages of the current methods and eliminates their complications. Our intramedullary device combines the stiffness of intramedullary nail devices, while still using a minimally invasive approach, and obviates the need for a secondary surgery associated with the use of external fixation. Our new intramedullary device improves on the TTC nail by sparing the subtalar joint, which has been proposed to allow for compensation of the malalignment associated with worsening tibiotalar arthritis, and thus its importance cannot be overlooked [9]. When compared to traditional open approaches and the use of screws and plates our technique and device have less periosteal stripping. Our open yet minimally invasive approach also allows for the ability to correct malalignment. We are able to accomplish this while minimizing the disadvantages of other techniques such as the risk of neurovascular injury with TTC nailing, the periosteal stripping associated with plating, the decreased biomechanical strength with the use of screws alone, and the inability to correct severe deformity with arthroscopic or mini open techniques.

Limitations of our study are inherent to the development of a new implant. This small study was able to demonstrate the ability of nail insertion; however, we did so in a cadaveric model with no way to measure the feasibility, reproducibility, effectiveness, and clinical outcomes in actual patients.

This study provides the foundation for future investigation. We propose expanding on this study with the use of fluoroscopy to aid in the application of the proximal interlock screws as well as the use of a guide wire and a flexible reamer for easier nail insertion. While the shape of the nail was designed to align the ankle joint in appropriate plantar/dorsiflexion as well as varus/valgus angulation using CAD tools and the standard saw bones model, the stiffness of the embalmed cadaveric specimens as well as the lack of proximal interlocking screws likely accounts for the plantarflexion seen in the lateral radiographs. Thus, further trials are still necessary to address this, likely with the use of thawed fresh frozen specimens. We also plan on perfecting our technique in order to decrease the size of the incision that was used in these initial trials in order to fully maximize the minimally invasive nature and goal of our new device. Currently, in the early stages of development, only computer simulated stress analysis was used to determine the feasibility of the lateral entry point as well as load tolerance of the construct; further in vitro mechanical testing would be required to confirm this and compare to the current implants available. While compression of the tibiotalar joint has not been found to decrease the time to union, it has been associated with decreased rates of nonunion; thus we are currently developing compression options for our new device [10]. Further cadaveric studies which implement these changes to the limitations that we noticed during our first attempt at using our new device in human tissue are warranted in order to progress our basic model for clinical use.

## 5. Conclusion

Our device represents a new method for isolated tibiotalar fusion which combines the advantages of current implants and techniques, while at the same time eliminating the common complications and disadvantages associated with them. With further testing and development, we believe our study provides the foundation to revolutionize tibiotalar arthrodesis.
